# Supplementary material for: Evidence for singular-phonon-induced nematic superconductivity in a topological superconductor candidate Sr0.1Bi2Se3
Source: Nat Commun. 2019 Jun 26;10:2802. doi: 10.1038/s41467-019-10942-2 (PMC6594966; doi:10.1038/s41467-019-10942-2)
Supplement: Supplementary file 1 — Supplementary Information [file 41467_2019_10942_MOESM1_ESM.pdf]

Supplementary Information for
Evidence for singular-phonon-induced nematic superconductivity in a topological superconductor candidate $Sr_{0.1}Bi_2Se_3$

Wang *et al.*

Supplementary Figures

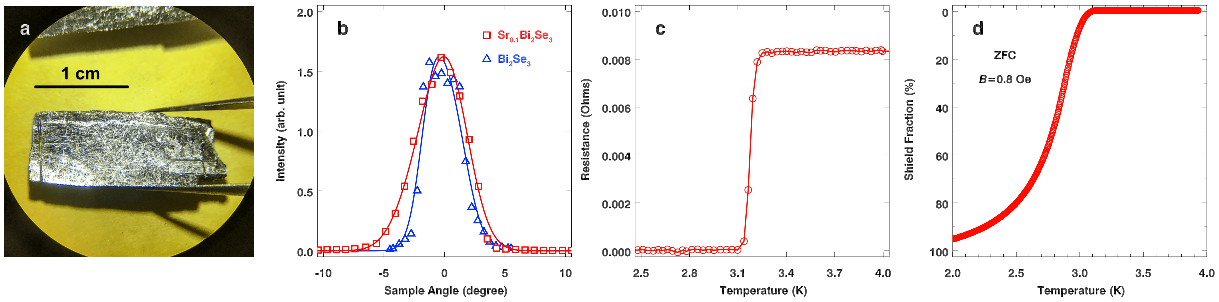

Supplementary Figure 1. **Sample characterisations.** **a**, A photograph of a $Sr_{0.1}Bi_2Se_3$ single crystal weighed 1.1 g used in the inelastic neutron scattering experiments. **b**, Rocking scans of $(1, 0, \text{-}5)$ Bragg peak for $Sr_{0.1}Bi_2Se_3$ (squares) and Bi_2Se_3 (triangles) obtained from neutron scattering experiments. The Bi_2Se_3 single crystal has a sample mass of 1.6 g. Lines through data are fits with Gaussian functions. Full width at half maximum is about 5 degrees for $Sr_{0.1}Bi_2Se_3$ and 4 degrees for Bi_2Se_3. Intensities are normalised for comparison purpose. **c**, Resistance in the *a*-*b* plane for one piece of single crystal cut from the $Sr_{0.1}Bi_2Se_3$ sample used in the neutron scattering experiment, showing a sharp superconducting transition with an onset at 3.2 K. **d**, Superconducting shield volume fraction estimated from the magnetization measured with an external field of 0.8 Oe under a zero-field cooling condition. A ratio over 95% suggests a large superconducting volume fraction in the sample, consistent with previous scanning tunneling microscopy results.[1]

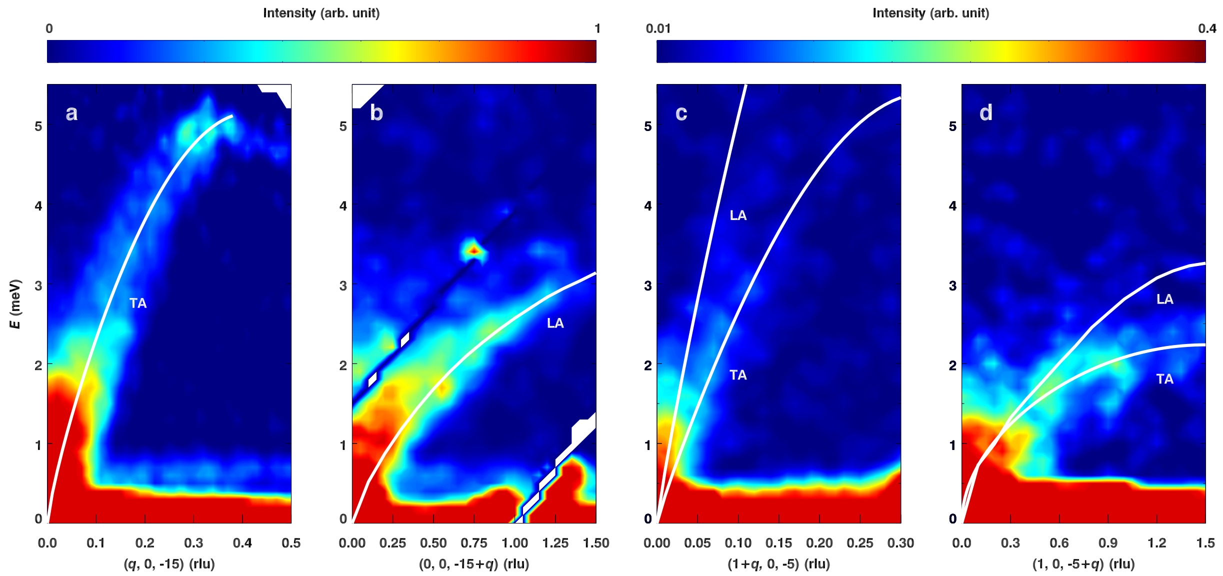

Supplementary Figure 2. **Phonon dispersions of a Bi_2Se_3 single crystal weighing 1.6 g, obtained on AMATERAS at $T = 17$ K. a** and **b**, phonon dispersing from $(0, 0, \text{-}15)$ along the [100] and [001] directions, respectively. **c** and **d**, phonon dispersing from $(1, 0, \text{-}5)$ along the [100] and [001] directions, respectively. The data plotted against the H direction in **a** were obtained by integrating the intensities in $(q, 0{+}K, \text{-}15{+}L)$ with a thickness of K and L ranging from -0.1 to 0.1 rlu, and -16 to -14 rlu, respectively. The data plotted in **b** were integrated with H=K=[-0.05,0.05] rlu, **c** with K=[-0.05,0.05] rlu and L=[-6,-4] rlu, and **d** with H=[0.95,1.05] rlu and K=[-0.05,0.05] rlu. E and q represent the phonon energy and reduced wave vector, respectively. TA and LA represent transverse and longitudinal acoustic modes, respectively. Those in **b** were integrated with $H = K = [-0.05, 0.05]$ rlu. E and q represent the phonon energy and momentum, respectively. TA, LA, and TO represent transverse acoustic, longitudinal acoustic and transverse optic modes, respectively. Solid lines are the dispersions obtained by fitting the energy scans at a series of q. The white streaks along the diagonal direction in **b** were due to the lack of detector coverage wherein.

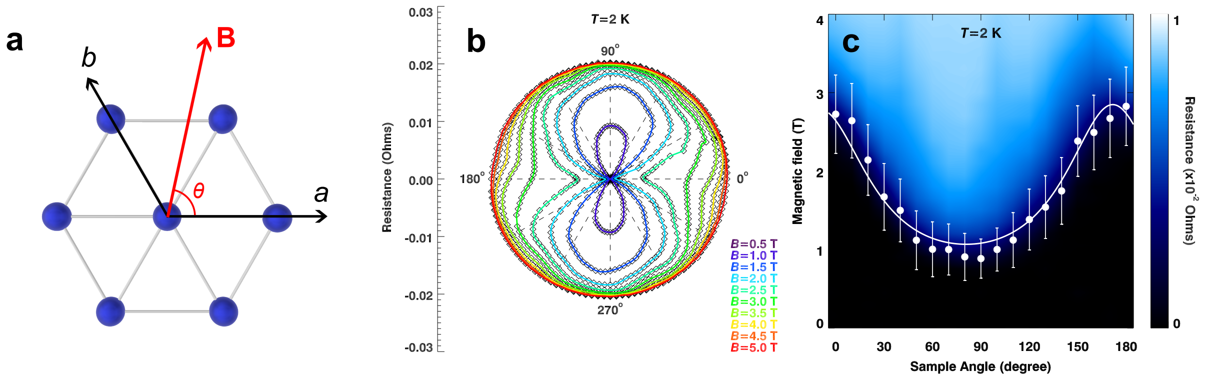

Supplementary Figure 3. **Two-fold nematic superconductivity in $\mathbf{Sr_{0.1}Bi_2Se_3}$.** **a**, Schematic of the experimental setup for the transport measurement. The sample was rotated within the a-b plane with an angle θ with respect to the magnetic field **B**. $\theta = 0$ roughly corresponds to the configuration where **B** is parallel to the a axis. Note that the a axis in this measurement was obtained by Laue X-ray and may not correspond to the [100] direction in the neutron experiment discussed in the main text. **b**, Angular dependence of the in-plane resistance measured at $T = 2$ K under different magnetic field strengths. **c**, In-plane angular dependence of the upper critical field H_{c2}. The H_{c2}s are determined to be the **B** value at which the slope of the resistance *vs.* **B** curve has a maximum. At each θ, the resistance as a function of **B** is plotted as the false-color contour. Lines through data are guides to the eye. Errors represent one standard deviation. These results clearly show a two-fold pattern, which breaks the in-plane three-fold rotation symmetry, indicating a p-wave nematic superconductivity in $Sr_{0.1}Bi_2Se_3$.

Reference

[1] Du, G. *et al.* Drive the Dirac electrons into cooper pairs in $Sr_xBi_2Se_3$. *Nat. Commun.* **8**, 14466 (2017).
